# Prevalence, Aetiological Agents, and Antimicrobial Sensitivity Pattern of Bacterial Meningitis Among Children Receiving Care at KCMC Referral Hospital in Tanzania

**DOI:** 10.24248/EAHRJ-D-16-00358

**Published:** 2018-04-01

**Authors:** Mohammed S Abdallah, Rune Philemon, Anaam Kadri, Ashley Al-Hinai, Aliasgher M Saajan, Joshua G Gidabayda, Gibson S Kibiki, Blandina T Mmbaga

**Affiliations:** a Department of Paediatrics and Child Health, Kilimanjaro Christian Medical Centre, Moshi, Tanzania; b Kilimanjaro Christian Medical University College, Moshi, Tanzania; c Kilimanjaro Clinical Research Institute, Moshi, Tanzania

## Abstract

**Background:**

Bacterial meningitis is an inflammation of the meninges that occurs in response to bacteria, causing a significant number of morbidity and mortality worldwide, especially in newborns and people living in low-income countries. Diagnosis of bacterial meningitis combines a high index of clinical suspicion and laboratory confirmation through cerebrospinal fluid (CSF) analysis. Despite antibiotic treatment, mortality remains high and many children end with long-term consequences, which include neurological deficits, hearing loss, and cognitive impairment.

**Objective:**

To determine prevalence, aetiological agents, and antimicrobial sensitivity pattern among children aged less than 13 years with bacterial meningitis at Kilimanjaro Christian Medical Centre (KCMC), Moshi, Tanzania.

**Methods:**

This was a hospital-based cross-sectional study carried out in the KCMC paediatric ward from December 2013 to May 2014 and from June 2015 to April 2016. In total, 161 children aged less than 13 years suspected of having meningitis were consecutively recruited. Each child submitted to a lumber puncture and CSF collected for microscopy, cultures, antimicrobial sensitivity testing, a latex agglutination test, and a polymerase chain reaction (PCR) test. PCR was run on 129 of the selected CSF samples. Data were collected using structured questionnaires and laboratory data sheet. Aetiological agents were identified, and antibiotic sensitivity was tested. Analyses were performed using SPSS version 20.0.

**Results:**

Overall, 24 children had confirmation of having acute bacterial meningitis. Of the 161 participants, Gram stain and culture identified 4 (2.5%) children; whereas, of the 129 samples tested using the PCR, infection was confirmed in 24 (18.6%) children. *Escherichia coli* (n=18) was the most common organism isolated followed by *Listeria monocytogenes* (n=3), *Streptococcus pneumonia* (n=1), *Group B Streptococcus* (n=1), and *Klebsiella* species (spp.) (n=1). With the exception of *Klebsiella* spp., the isolated organisms were sensitive to the following commonly used antibiotics: ampicillin, chloramphenicol, gentamycin, and cephalosporin.

**Conclusion:**

PCR yielded more organisms. *E. coli* was the most common organism and was sensitive to the empirically used antibiotics for treatment of bacterial meningitis tested in our study.

## INTRODUCTION

Bacterial meningitis is an inflammation of the meninges that occurs in response to bacteria, causing a significant number of morbidity and mortality throughout the world, especially in newborns and people living in low-income countries. Those with infections caused by antimicrobial-resistant bacteria have increased risk.^1^

Even with the challenge of under-reported cases, the mortality associated with neonatal meningitis in lowand middle-income countries varies between one-third and two-thirds of confirmed cases of meningitis.^2^ About 70% of the survivors develop chronic conditions like cerebral palsy, deafness, blindness, seizure disorders, and mental retardation.^3,4^

Clinical features with a high index of suspicions for meningitis vary depending on age; some are non-specific as some overlap with other conditions or diseases, such as sepsis and severe malaria. In neonates and young children, symptoms include poor feeding, lethargy, irritability, apnea, listlessness, apathy, fever, hypothermia, seizures, jaundice, bulging fontanelle, pallor, shock, hypotonia, a shrill cry, hypoglycemia, and intractable metabolic acidosis; whereas, in infants and children the presentation includes nuchal rigidity, opisthotonos, bulging fontanelle, convulsions, photophobia, headache, alterations of the sensorium, irritability, lethargy, anorexia, nausea, vomiting, coma, fever, or hypothermia.^5^

Diagnosis of bacterial meningitis combines a high index of clinical suspicion and laboratory confirmation through analysis of the cerebral spinal fluid. Identification of the type of bacteria causing the disease is either by Gram stain, cultures, a latex agglutination test, or by a polymerase chain reaction (PCR) test. In the absence of cerebrospinal fluid (CSF) analysis results, empiric antibiotic treatment is recommended to cover the major pathogens due to the high mortality of this disease.

The aetiological agents of meningitis vary from one place to another, as does their antibiotic susceptibility. In Tanzania, the most common aetiology previously reported in children include *Streptococcus pneumoniae, Haemophilus influenzae*, and *Klebsiella* species (spp.).^6,7^ However, *Salmonella* spp., *Escherichia coli*, and *Neisseria meningitidis* have also been reported, but at lower rates.^7^ A study in Tanzania showed that among children admitted with fever, meningitis contributed to 0.2% and malaria to 10.5%^8^ of the cases, while another study showed a prevalence of suspected meningitis being 9.6%, with bacteriological confirmed meningitis of 5.6%, 61% of those cases were children less than 12 months of age.^7^ Despite following the recommended management for treating bacterial meningitis, we still find some children ending up with serious neurological consequences and death, suggesting the possibility that the causative microbes in our setting might have developed resistance to the antimicrobial agents that we are using, specifically, ampicillin, chloramphenicol, and ceftriaxone.^5^ A study in Dar es Salaam reported mortality for children, isolated *S. pneumoniae, H. influenzae*, and *Klebsiella* spp. each represented 22.5% of the deaths.^7^ In 2009 and 2013, the Expanded Program of Immunization (EPI) introduced the *H. influenzae* (Hib) vaccine and pneumococcal conjugate vaccine (PCV), respectively, to prevent *S. pneumoniae* and *H. influenzae*, which are among the major causes of meningitis in children. In Tanzania, the vaccination status for these vaccines shows good coverage for the last doses of Hib3 (97%), PCV3 (96%), polio (93%), and measles vaccination (90%) as reported in 2016.^9^

At the Kilimanjaro Christian Medical Centre (KCMC) paediatric ward, meningitis is one of the top 10 diseases for which children are admitted; however, no data are available on either its mortality patterns or the microbiological pattern. To date, the aetiology and sensitivity of antimicrobials used to treat meningitis has not been systematically studied. Since bacterial meningitis makes up a significant proportion of disease morbidity and mortality and is among the major health problems in our setting, identification of the causative bacteria and pattern of antibiotic sensitivity is a priority for the referral and teaching hospital. Furthermore, for a serious infection like bacterial meningitis, it is necessary for periodic sensitivity assessment of the causative agents, especially where there is limited rapid investigation to guide clinicians on proper antibiotic treatment selection. Therefore, we aimed to determine the prevalence, aetiological agents, and antimicrobial sensitivity pattern of bacterial meningitis among children receiving care at KCMC who are suspected of having bacterial meningitis. The findings of this study may help to improve rational drug use in managing meningitis and, hence, in reducing morbidity and mortality.

## METHODS

### Study Design

This was a cross-sectional hospital-based study conducted at KCMC between December 2013 and May 2014, and then extended to June 2015 through April 2016. The study was conducted within the paediatric ward at KCMC hospital, which is both a referral hospital and a research and teaching hospital. It serves the Northern zone of Tanzania, which includes six regions: Kilimanjaro, Arusha, Tanga, Manyara, Singida, and Dodoma, with a catchment of more than 10 million people. In the paediatric ward there are three units, which together have 16 rooms, giving the ward a total capacity of 91 beds.

### Study Population

All children aged less than 13 years, clinically suspected to have meningitis, and admitted into the paediatric ward at KCMC during the study period were included in this study. Children who had contraindication to lumber puncture and those whose parent/guardian refused to provide consent were excluded from the study.

### Case Definitions

A confirmed case of meningitis was defined as one of the following: bacterial isolation from a CSF culture positive, Gram stain, latex agglutination test, or PCR test.

### Sample Size Estimation

The minimum sample size was estimated using a formula by the Survey System (Creative Research Systems, Sebastopol, CA, USA) and the Joint WHO and Directorate-General for International Cooperation (1988) expressed as sample size (SS) = Z^2^ (P)(1-P)/ε^2^, where, Z = value (1.96 for 95% confidence level [CI]). A prevalence (P) of 9.6% for suspected bacterial meningitis was selected based on a study done in Tanzania by Kalokola et al., 2007^7^ and ε = minimal tolerable error at 95% CI, expressed as a decimal (0.05). The minimum estimated sample size was 133 participants. The study used a convenience sampling technique where all children admitted with a diagnosis of meningitis were voluntarily enrolled after consent was given by parents/caretakers.

### Data Collection

After enrolment, interviewers administered a questionnaire to collect data on the age, sex, presenting complaint, relevant signs elicited, antibiotic pretreatment, immunization status, HIV serostatus, and socioeconomic status of each patient. The study team used a data sheet to record information from the laboratory tests, including blood glucose, CSF macroscopic, Gram stain, CSF culture, drug sensitivity, latex agglutination test, and PCR results.

### Sample Collection

For older children, raised intracranial pressure was ruled out through fundoscopy. For neonates and infants, especially those with an open fontanel and acute onset illness, lumbar puncture was done independent of fundoscopy using 23-gauge spinal needles. Two to 3 ml of CSF was collected aseptically in a sterile test tube. All specimens were labelled and immediately sent to the Kilimanjaro Clinical Research Institute (KCRI) biotechnology research laboratory situated within KCMC campus.

Upon arrival, each specimen was examined macroscopically to determine whether the sample was clear, slight turbid, cloudy, purulent, or bloody. All CSF samples, except those that appeared turbid, were centrifuged for 15 minutes; 500 μl was collected in cryotubes and stored in a freezer at −80 degrees Celsius for later PCR analysis. All CSF samples were immediately processed using Gram stain and culture analysis.

### Laboratory Analysis

Laboratory analysis was done at the KCRI biotechnology research laboratory. The sediment was cultured using standard and Gram stain techniques. The sediment of centrifuged CSF from the sterile bottle was inoculated using a sterile loop onto chocolate, blood, and MacConkey agar plates (Becton Dickinson [BD] International Branch of Becton Dickinson BV, Belgium). All isolates were identified on the basis of their colony, morphology, culture characteristics, and biochemical tests following standard procedures. The susceptibility patterns of the isolates were determined by diffusion technique according to the Clinical Laboratory Standard Institute (CLSI).^10^ Susceptibility to antibiotics was defined and categorised by Rodloff et al^11^ as: susceptible (S), when an organism is inhibited in vitro by a concentration of the drug that is associated with a high likelihood of therapeutic success; intermediate (I), when an organism is inhibited in vitro by a concentration of the drug that is associated with an uncertain therapeutic effect; and resistant (R), when an organism is inhibited in vitro by a concentration of the drug that is associated with a high likelihood of therapeutic failure.

DNA extraction of the stored CSF samples was done using QIAamp DNA Mini Kit (QIAGEN, Hilden, Germany) according to manufacturer's instructions. Bacteria were detected by using a real-time multiplex quantitative PCR on a ViiA 7 Real-Time PCR System (Thermo Fisher Scientific, Waltham, Massachusetts, USA), using the Fast track Diagnostics (FTD) bacterial meningitis and neonatal meningitis kits (Fast Track Diagnostics, Junglinster, Luxembourg). Each of these two assays were developed to detect three different targets: FTD bacterial meningitis detects *N. meningitidis, S. pneumoniae*, and *H. influenzae* and FTD neonatal meningitis detects *S. agalactiae/Group B Streptococcus* (GBS), *L. monocytogenes*, and *E. coli.*

### Data Processing and Analysis

Data were entered and analysed using Statistical Package for Social Science (SPSS), version 20 (IBM Corp., Armonk, New York, USA). Data summarization was done using mean and standard deviation for continuous variables and frequency and percentage for the categorical variables. Data presentations were done using bar charts, graphs, and tables.

### Ethical Consideration

Ethical approval for the study was obtained from the Institutional Ethical Review Board of Kilimanjaro Christian Medical University College (KCMUCo). Parents/caretakers were requested to read and sign a written informed-consent document prior to enrolment. Participation was voluntary, and parents/guardian had the right to withdraw their children from participating in the study at any time. Those parent/guardians who refused to provide consent for their children to participate in the study received equal clinical management.

## RESULTS

**FIGURE 1.**
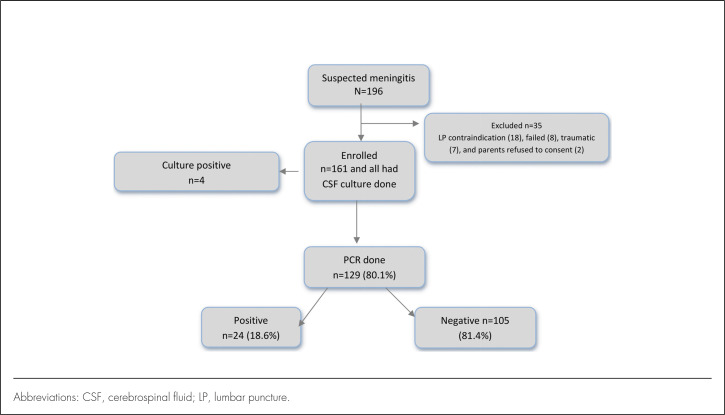
Description of Study Participants Enrolled in Meningitis Study

Abbreviations: CSF, cerebrospinal fluid; LP, lumbar puncture.

**FIGURE 2.**
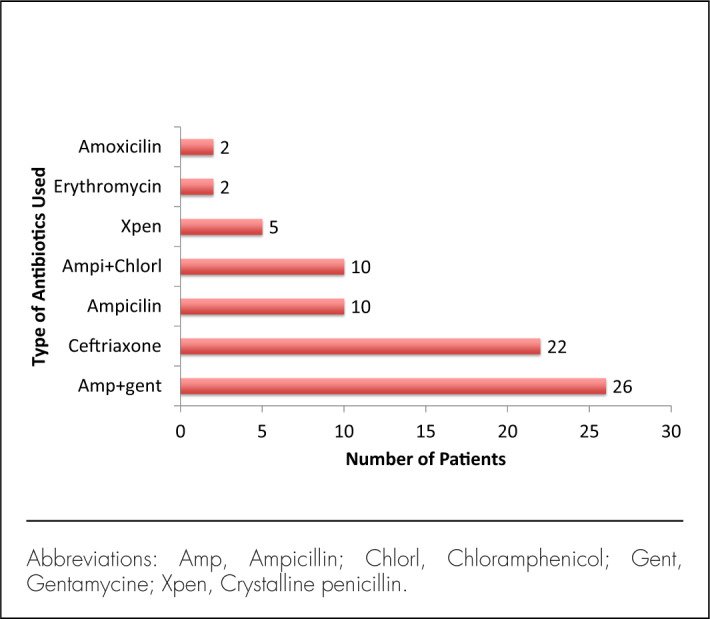
Types of Antibiotic Used

Abbreviations: Amp, Ampicillin; Chlorl, Chloramphenicol; Gent, Gentamycine; Xpen, Crystalline penicillin.

During the study period, 196 children were hospitalized with a clinical diagnosis of meningitis. After applying exclusion criteria, 35 children were excluded, and the remaining 161 children were enrolled in this study (**Figure 1**). The median age at enrolment was 8 months, with an age range of birth to 13 years, and most of participants were male (61.5%). Of the enrolled participants, slightly more than half of all study participants (52.2%) reported having used antibiotics prior to admission (**Table 1**). The majority of antibiotics prescribed at peripheral hospitals prior the present admission included an ampicillin and gentamycin combination (n=26, 34.2%), ceftriaxone (n=22, 29%), an ampicillin and chloramphenicol combination (n=10, 13.2%), or ampicillin alone (n=10, 13.2%) (**Figure 2**); other antibiotics recorded were metronidazole, cotrimoxazole, and erythromycin.

**TABLE 1.** Baseline Characteristics of the Participants (N=161)

Baseline characteristics	n	%
Age group, months		
<1	51	31.7
1–12	53	32.9
>12	57	35.4
Median (range); months	8.0 (1–153)	
Sex		
Female	62	38.5
Male	99	61.5
Antimicrobial use		
No	77	47.8
Yes	84	52.2
Comorbidities on admission		
Acute Flaccid Paralysis	1	0.6
Acute Lymphoblastic Leukemia	1	0.6
Coarctation of Aorta	1	0.6
Febrile convulsion	2	1.2
Head Injury	1	0.6
Hydrocephalus	1	0.6
Impetigo	2	1.2
Juvenile Idiopathic Arthritis	1	0.6
Metabolic Alkalosis	1	0.6
Neonatal Sepsis	51	31.7
Otitis Media	4	2.5
Pneumonia	14	8.7
Presumptive HIV	2	1.2
Septicemia	42	26.1
Severe Anemia	1	0.6
Severe Malaria	24	14.9
SOL	1	0.6
URTI	9	5.6
UTI	2	1.2

Abbreviations: SOL, space occupying lesion; URTI, upper respiratory tract infection; UTI, urinary tract infection.

**FIGURE 3.**
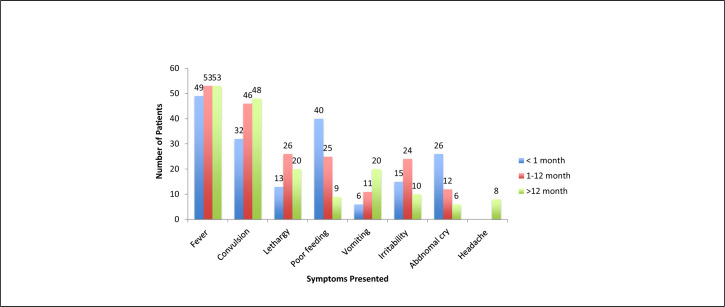
Symptoms Presented by Age Groups

**FIGURE 4.**
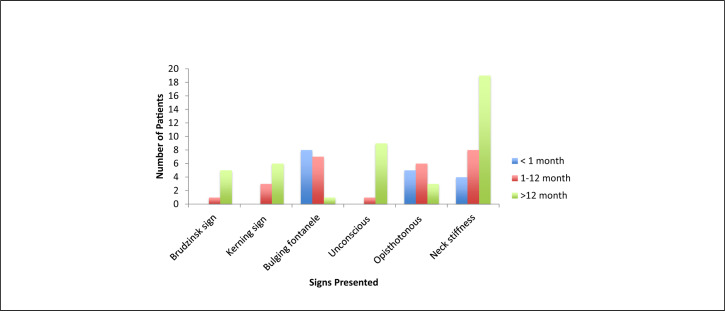
Signs of Meningeal Irritation by Age

Of the 161 study participants, a vast majority 148 (91.9%) had a fever with a body temperature of >38°C at enrolment, 101 (62.7%) presented with fever for less than 3 days and 127 (78.3%) presented with seizures majority being neonates. Slightly less than half (46.0%) of the children presented with a symptom of reduced/poor feeding (**Figure 3**). Slightly more than one-third (36.6%) of the children demonstrated lethargy/drowsiness. Over a quarter (27.3%) of children suspected to have meningitis had an abnormal cry at presentation. The most presented sign in older children was neck stiffness (**Figure 4**).

CSF was cultured for all 161 participants. From these cultures, a total of 4 (2.5%) CSF samples had bacteria growth. Gram stains detected 4 bacteria: 1 Gram-negative bacilli, 1 Gram-negative rods, and 2 Gram-positive cocci. A Wellcogen latex agglutination method was conducted with 81 samples. Only 1 CSF sample showed agglutination test positive toward GBS.

**FIGURE 5.**
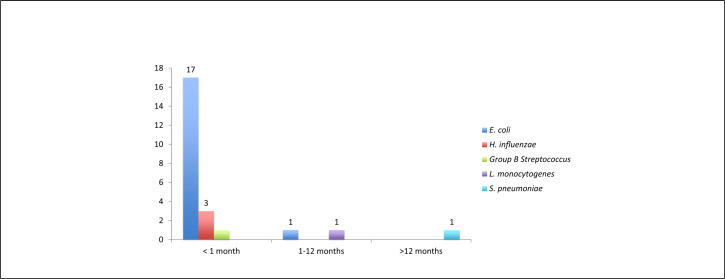
Isolated Organisms by Age Groups

PCR testing was done in 129 out of 161 (80.1%) samples collected from all participants, due to shortage in reagents for the first recruitment period. PCR positive test was detected in 24 out of 129 (18.6%) children with meningitis (**Table 2**). *E. coli* was the most common isolated bacteria (n=18) followed by *Listeria monocytogenes* (n=3), *S. pneumonia* (n=1), GBS (n=1) and *Klebsiella* spp. (n=1) (**Figure 5**). The most affected age group with *E. coli* isolates (n=17) were neonates aged less than 28 days of life. Based on Gram stain and culture results, *E. coli*, GBS, *Klebsiella* spp., and *S. pneumonia* were each observed in 1 child.

**TABLE 2.** Distribution of Study Participants According to Methods Used to Identify the Aetiological Agents of Meningitis (N=161)

Method	Results n (%)		Total n
	Positive	Negative	
Gram stain	4 (2.5)	157 (97.5)	**161**
Culture	4 (2.5)	157 (97.5)	**161**
Wellcogen agglutination	1 (1.2)	80 (98.8)	**81**
PCR	24 (18.6)	105 (81.4)	**129**

Abbreviation: PCR, polymerase chain reaction.

A total of 9 antibiotics were used for determining the antimicrobial sensitivity pattern. All of the isolates were sensitive to ceftriaxone. *E. coli* was not sensitive to cotrimoxazole and cloxacillin. *S. pneumoniae* was not sensitive to cotrimoxazole, however, it was intermediately sensitive to cloxacillin. *Klebsiella* spp. was not sensitive to ampicillin, cloxacillin, gentamicin, cotrimoxazole, amoxicillin/clavulanic acid, and erythromycin, but was intermediately sensitive to chloramphenicol (**Table 2**).

**FIGURE 6.**
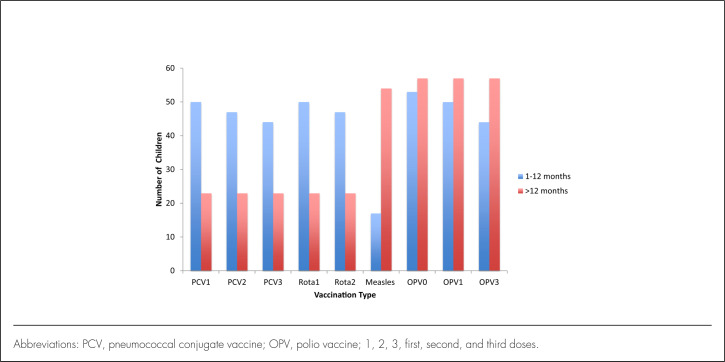
Vaccination Status by Age Groups

Abbreviations: PCV, pneumococcal conjugate vaccine; OPV, polio vaccine; 1, 2, 3, first, second, and third doses.

A majority of children in this study were reported as having received vaccinations according to their age, with almost 95% coverage for measles; only 3 (5%) children above 12 months had not received a measles vaccination (**Figure 6**). In contrast, only 23 children in the same age group received the PCV and rotavirus vaccines, and none of the neonates had received any vaccination prior to admission.

## DISCUSSION

The study of meningitis in children was aimed at identifying the common causative bacteria and their antibiotic sensitivity in children with suspected meningitis. In our study, the rate of identification was 2.5% using conventional methods of CSF analysis, which included Gram stain and culture. The observed result was in accordance with prevalence reported in Ilesa, Nigeria (1.6%)^12^; Tehran, Iran (2.9%)^13^; and Ghana (3.3%).^14^

This prevalence is much lower than what was reported in Kenya (17.9%),^15^ Nigeria (15.3%),^16^ Bangladesh (14.4%),^17^ and France (15.0%),^18^ which used similar conventional methods. The difference in results found between our study and the others may be due to their exclusion of patients who had received antibiotics before admission, which could have resulted in having a higher yield of live bacteria using both the culture technique and advanced techniques such as the latex agglutination and PCR tests. In our study, slightly more than half (52.2%) of all study participants reported having used antibiotics prior to admission at KCMC. The use of antibiotics was either the result of self-medication, parent/guardian administration, or empiric treatment according to the Integrated Management of Childhood Illness (IMCI) guidelines^19,20^ used in the lower health facilities the patients were referred from. The IMCI guidelines recommend giving antibiotics to all patients with severe febrile illness before referral. This could have contributed to low number of isolates by culture.

In our study, the prevalence increased when CSF tested by PCR. This supports the higher sensitivity of PCR in CSF examination for identification of meningitis. Similar higher yield reported in France (15.0%), where PCR was also used.^18^ The higher sensitivity of PCR can be demonstrated by the increase in yield when specimens from our study were run through PCR against common bacteria causing meningitis in neonates and children—specifically, GBS, *E. coli, L. monocytogenes, H. influenza* type b, *N. meningitidis*, and *S. pneumonia*, which substantially increased the CSF yield to 24 (18.6%) of 129 tested samples; however no *N. meningitidis* was isolated. This was similar to a study of CSF samples by Ceyhan et al in Turkey, whereby PCR analysis was by far the most reliable method of confirming acute bacterial meningitis, accounting for 243 (59.6%) of the 408 CSF samples tested, while CSF cultures only confirmed 41 (10%).^21^ In Burkina Faso, researchers had similar findings showing that PCR is both reliable and more sensitive than other conventional methods for detecting the aetiological agents of meningitis.^22^ In this study, the most common aetiological agent was *E. coli* (n=18). The predominance of *E. coli* was similar to what was reported in Nigeria^3^ and Kenya^15^ where both studies reported *E. coli* being the most common bacteria isolated.^3,15^ However, in Taiwan^23^ and Niger state,^24^ *E. coli* was the second most common bacterial isolate; it has also been infrequently reported in Bangladesh,^17^ Nigeria,^12,25^ Ghana^14^ and in Dar es Salaam, Tanzania.^7^ The identification of higher predominance of *E. coli* in the current study may also be related to the young age group in which *E. coli* is predominant.

In neonates, *L. monocytogenes*, a food-borne disease, was isolated from three neonate samples. *L. monocytogenes* is counted as one of the infectious causes of sepsis in neonates. The disease is common in individuals with low immunity, including pregnant women, which makes neonates more susceptible. The diagnosis is challenging with convectional culture methods; PCR makes the isolation possible, as shown in previous case studies in neonates.^26,27^ If not recognized and treated properly, *L. monocytogenes* can lead to meningitis and hydrocephalus, as complication^27^; therefore, this bacteria should be considered in neonates with sepsis and meningitis.

The second bacteria identified by conventional methods in a child aged above 12 months was *S. pneumonia* (n=1). *S. pneumonia* is one of the most common bacteria that cause meningitis in Africa, as shown in studies in Ghana,^14^ Nigeria,^12^ and Dar es Salaam, Tanzania.^7^ The introduction of the Hib and PCV vaccines in 2009 and 2013, respectively, in the Tanzania EPI programme may have played a role in the low detection of *S. pneumonia* and *H. influenza* observed in this study. In this study, also we observed good vaccination coverage according to age, similar to national coverage levels, with the except of measles, where the coverage was higher (95%) compared to national estimate (90%).^9^

In our study, all children investigated by PCR had received 100.0% immunization as per recommended EPI schedule according to age. It has been demonstrated in other settings that with the introduction of *H. influenza* vaccination, the prevalence of *H. influenza* type b as a cause for meningitis goes down, this was as exemplified in the studies done in Kenya^28^ and Uganda.^29^

Ampicillin was effective against *S. pneumonia*, unlike the findings from studies done in Nigeria^16,30^ that showed *S. pneumonia* to be resistant to ampicillin. Resistance of *E. coli* to ampicillin was reported in studies done in Bangladesh^17^ and Ghana.^14^ Although most of the remaining drugs tested were shown to be effective, it is difficult to draw conclusions on the antibiotic sensitivities against the aetiological agents causing meningitis because of the small number of bacterial isolates on CSF cultured.

### Limitations

The study was done in a tertiary care hospital and, logistically, it was not possible to conduct investigations for various funguses, viruses, and tuberculosis. It was not possible to conduct PCR tests on all collected CSF; however, cultures were done in all samples. The positive yield for cultures was too small to make proper inference on susceptibility. The team was unable to follow up in the final outcomes of children, which would have shown the effect of management.

## CONCLUSIONS AND RECOMMENDATIONS

Bacterial meningitis is an important clinical problem in our hospital setting, with *E. coli being* the most common aetio-logical bacteria identified in neonates. The effectiveness of a meningitis diagnosis can be improved with a PCR test. In this study, it was difficult to draw conclusions on the antibiotic sensitivities due to low yield by culture; however, other commonly employed first-line antibiotics were effective against the isolated bacteria, excluding *Klebsiella* spp. We believe that monitoring the aetiological agent causing meningitis and updated information of their antibiotic susceptibility pattern is required.

**TABLE 3.** Antimicrobial Sensitivity Patterns of the Isolated Organism (N=4)

	Antibiotics Sensitivity								
Isolated Organisms	Ampicillin	Cloxacillin	Ceftriaxon	Gentamycin	Chloramphen	Amoxiclav	Cotrimoxazole	Erythromycin	Ciprofloxacin
*E. Coli*	S	R	S	S	S	S	R	S	S
GBS	S	S	S	S	S	S	I	S	S
*S. pneumoniae*	S	I	S	S	S	S	R	S	S
*K. pneumoniae*	R	R	S	R	I	R	R	R	S

Abbreviations: Amoxiclav, amoxicillin/clavulanic acid; *E., Escherichia*; GBS, Group *B Streptococcus*; I, intermediate resistance; *K., Klebsiella*; S, sensitive; *S., Streptococcus*; R, resistant.

In light of the discrepancy between the yield of bacteria using PCR and cultures, more sensitive routine testing of patients suspected to have meningitis is needed in order to curb morbidity and reduce complications that can result from untreated meningitis. As PCR testing is still unaffordable for most patients and unattainable by most health facilities, rapid diagnostic tests with higher sensitivity than cultures might be a viable option for low- and middle-income countries, if they can be shown to work in our settings.
